# Scrambler Therapy for the Treatment of Chronic Post-Mastectomy Pain (cPMP)

**DOI:** 10.7759/cureus.1378

**Published:** 2017-06-21

**Authors:** Thomas Smith, Andrea L Cheville, Charles L Loprinzi, Denise Longo-Schoberlein

**Affiliations:** 1 Palliative Medicine Program, Johns Hopkins Sidney Kimmel Comprehensive Cancer Center; 2 Physical Medicine and Rehabilitation, Mayo Clinic and Foundation, Rochester, MN, USA; 3 Medical Oncology, Mayo Clinic and Foundation, Rochester, MN, USA

**Keywords:** post mastectomy pain, scrambler therapy, neuropathic pain, breast cancer, survivorship

## Abstract

Chronic post-mastectomy pain (cPMP), including post-lumpectomy pain, is common with no established ways of treatment. We treated three consecutive patients referred with cPMP with scrambler therapy (ST), a non-invasive electrical neurocutaneous stimulation. Treatment was given across the area of pain following the dermatomes for 45 minutes daily, for several consecutive days until relief, and then was repeated as needed. The ST MC5A device synthesizes 16 different waveforms that resemble action potentials, delivered to the surface receptors of the c-fibers, to send “non-pain” information along the damaged pathways to reduce central sensitization. All three had marked (over 75%) and sustained (months) reduction of allodynia, hyperalgesia, and pain. All reported marked improvements in their quality of life and normal function. One woman was able to stop chronic opioid use. No side effects were observed.

Scrambler therapy is a promising way to relieve cancer and other types of neuropathic pain, and may be helpful in cPMP. Further prospective trials are warranted.

## Introduction

Chronic post-mastectomy pain (cPMP) is defined as “chronic pain in the anterior aspect of the thorax, axilla, and/or upper half of the arm beginning after mastectomy or quadrantectomy and persisting for more than three months after the surgery” [1-2]. The pain may be due to damage to the intercostobrachial nerve, the lateral cutaneous branch of the second intercostal nerve that is often resected at mastectomy and damaged in 80%-100% of mastectomy patients who undergo axillary dissection. This direct nerve damage explains the allodynia (painful sensation on normal touch), hyperalgesia, and pain. Post-mastectomy pain occurs in about half of all breast cancer patients [3] with an increased incidence among those who had chronic preoperative pain [4]. In one of the most comprehensive surveys, it was shown that 47% of women reported post-mastectomy pain, and the pain was severe in 13% of the total sample [5].

There are few evidence-based treatments for this clinical problem [6]. Some established methods for prevention include preoperative bupivacaine in the epidural space, memantine, gabapentin, or venlafaxine, but these have not been evaluated for treatment of established cPMP.

Scrambler therapy (ST) is an FDA-cleared treatment for neuropathic pain supported by multiple trials [7]. Two dozen published ST reports have covered over 800 patients with non-CMP types of cancer pain, chemotherapy-induced neuropathic pain, back pain, and post-herpetic neuropathy. The machine synthesizes 16 different electronic waveforms which are hypothesized to be perceived by surface C-fiber receptors, and travel along the C-fibers as “non-pain” information in dermatomes adjacent to the painful areas [8]. Over time, repeated doses of ST appear to reset the perceived pain process.

We recently treated two cPMP patients and one post-lumpectomy patient with ST and all had relief of established pain; we present them to stimulate further prospective research. To our knowledge, these are the first reported cases of cPMP treated with ST.

Investigational Review Board approval is not required if three or fewer cases are reported. Signed permissions from all three participants are on file in the office of the corresponding author. 

## Case presentation

### Case 1

**Figure 1:**
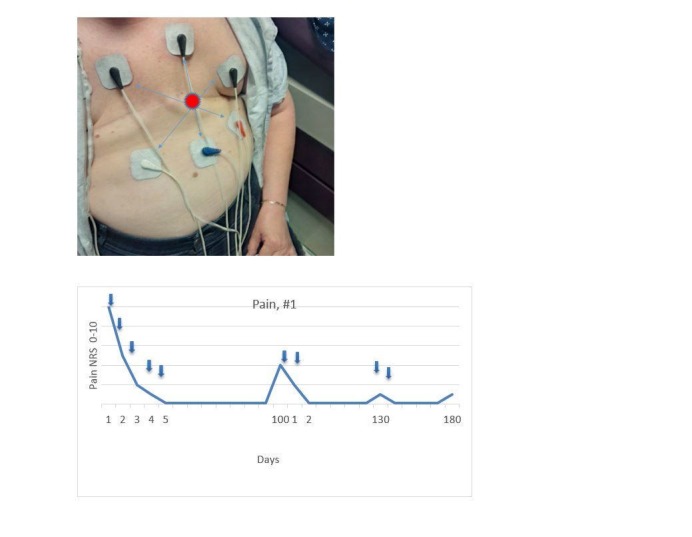
Electrode Placement, Case 1 Placement of scrambler therapy electrodes, Case 1. Electrodes are paired to each other as shown by the arrows. The major pain site is indicated in the center. Pain response is shown below.

 A 60-year-old woman had had a bilateral mastectomy two years ago, with no subsequent chemotherapy or radiation. She described her cPMP as “a barbed wire bra” on the front with several more painful spots, noted as the circle in Figure 1. Before treatment, her pain was 8-10/10, which decreased to 0-2/10 after five treatments, each lasting 45 minutes. She resumed painting and had a relatively more normal life, and was able to taper off and eventually stop her opioids. Her pain returned four months later and was successfully retreated (Figure 1). Her cancer subsequently metastasized to bones and became her dominant issue.

### Case 2

**Figure 2:**
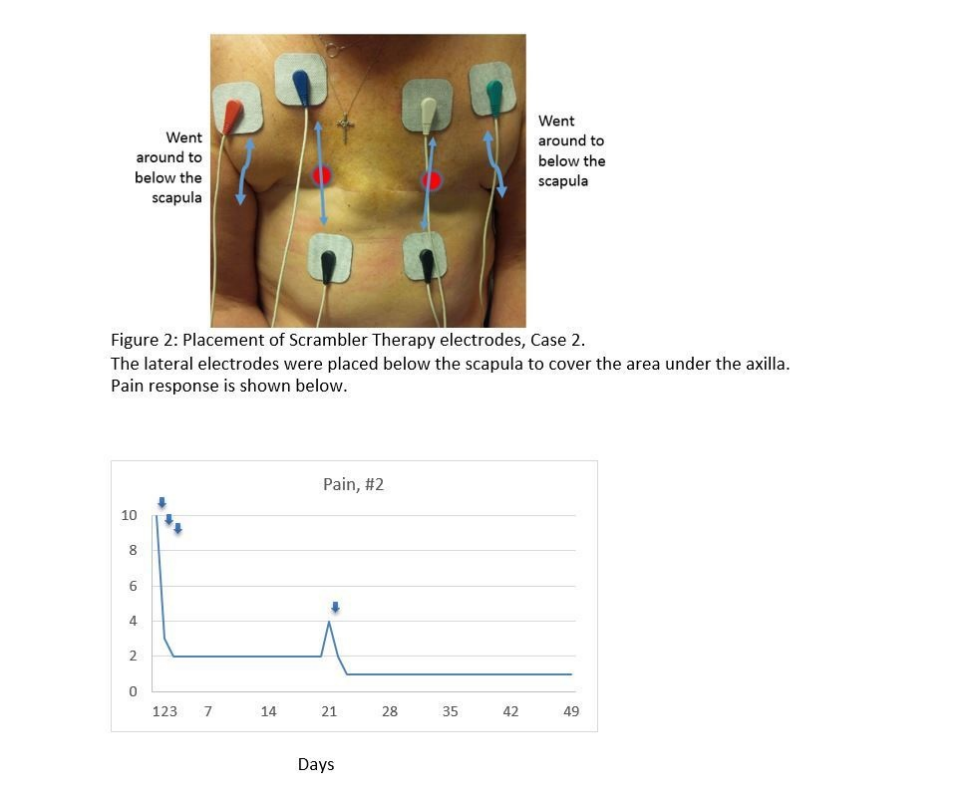
Placement of Scrambler Therapy Electrodes, Case 2

 This 58-year-old woman had deep and aching post-mastectomy pain after mastectomy and chemotherapy, which was sharp where the circles are noted in Figure 2, for almost a year. Her pain was 10/10 before treatment; she obtained relief during treatment and her pain decreased to 0-1/10 after two 45-minute treatments. Her pain returned one month later; three treatments reduced her pain from 4/10 to 0/10, which lasted for several months. She noted, as did Patient 1, a marked improvement in her quality of life. She continues on single intermittent treatments every 30-90 days (Figure 2).

### Case 3

**Figure 3:**
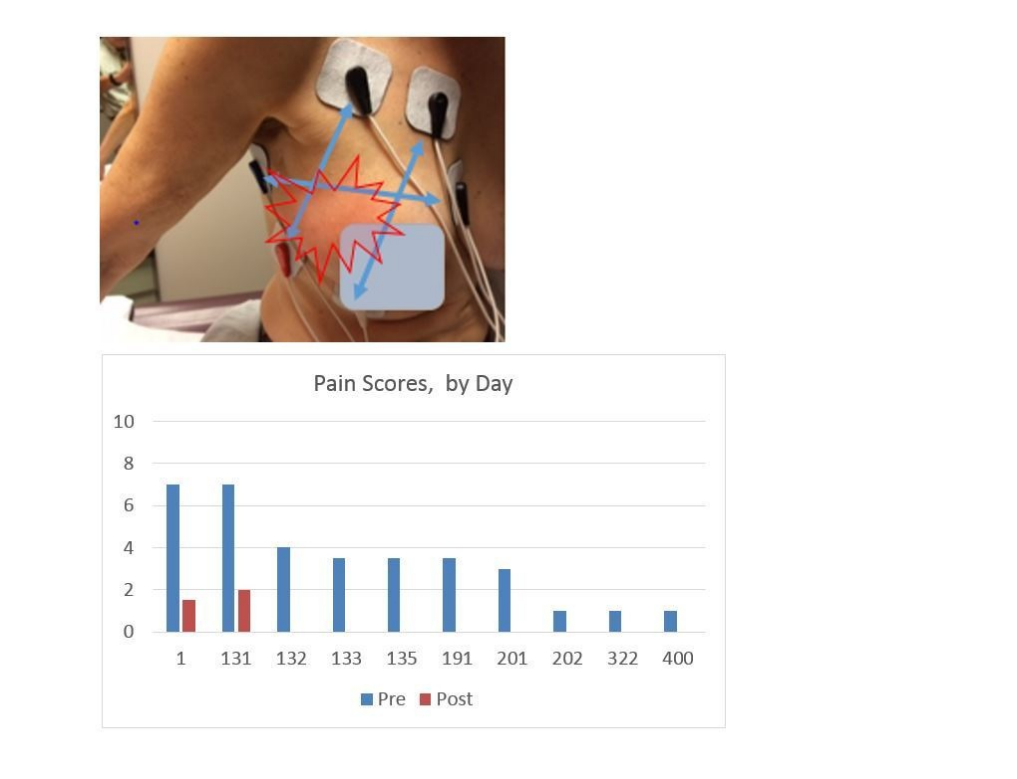
Electrode Placement, Case 3 Allodynia disappeared 10 minutes into treatment on Day 1. Pain response is shown below. The “post” treatment score was reduced from 7 to 1.5 Day 1, 7 to 2 Day 131, and then 0 after every other treatment. Her last treatment was Day 201. Day 2-130 no ST therapy was available.

 This 69-year-old woman received neoadjuvant docetaxel, trastuzumab, and pertuzumab with dramatic response and no untoward side effects. She then had a standard lumpectomy and lymph gland dissection followed by standard breast radiotherapy. Some weeks afterwards, she developed whole-breast allodynia with subsequent redness, swelling, and edema; marked reduction in shoulder mobility; and marked burning pain throughout the breast and axilla. There was no documented recurrence, and her pain persisted for a year, worsening to 10/10 worst pain and at best 6/10 deep aching pain. Treatment was given first to the breast, following the dermatomes across the breast, then to the axilla. After one treatment her allodynia was markedly better, as was the pain. She was retreated with several more treatments over the next months and is markedly better, back at work, able to wear normal clothes, and hasfull mobility of the shoulder and axilla (Figure 3).

## Discussion

We report the first use of scrambler therapy to treat cPMP and post-lumpectomy pain. Scrambler therapy appears to improve pain scores and enabled one patient to reduce her opioids with the intent of stopping. New CDC guidelines recommend against long-term opioid treatment for chronic pain and call for new, safer approaches to pain management. The post-lumpectomy patient may have had a complex regional pain syndrome that manifested as pain, redness, edema, and allodynia and worsening over time after an insult [9] and for which ST has recently been reported to be uniquely helpful [10].

These treatments were all performed by a physician (TS) or advanced practice nurse (DL-S), but can be performed by trained technicians, nurses, and physical or occupational therapists under supervision, and with knowledge of dermatomes. In our experience, insurance has typically covered the time of the clinician performing the procedure, but not a procedure code like a spinal injection. Scrambler therapy was cleared by the Food and Drug Administration in 2009 for safety.

## Conclusions

Better treatments are needed for chronic pain such as cPMP. Scrambler therapy appears to hold promise, as it does in chemotherapy-induced neuropathy, failed back syndrome, post herpetic neuropathy, low back pain and other conditions. Prospective trials are indicated to further evaluate this treatment modality.
